# Tolerance Mechanisms and Removal Efficiency of *Chlorella pyrenoidosa* in Treating 3-Fluorophenol Pollution

**DOI:** 10.3390/metabo14080449

**Published:** 2024-08-15

**Authors:** Min Li, Zhenfang Shang, Yonglan Ma, Huijun Zhao, Zhijing Ni, Zhaojun Wei, Xiu Zhang

**Affiliations:** 1 School of Biological Science and Engineering, North Minzu University, Yinchuan 750021, China; limin_nx@nmu.edu.cn (M.L.); 20227591@stu.nmu.edu.cn (Z.S.); 20237620@stu.nmu.edu.cn (Y.M.); zjwei@hfut.edu.cn (Z.W.); 2007057@nmu.edu.cn (X.Z.); 2 Ningxia Key Laboratory of Microbial Resources Development and Applications in Special Environment, Yinchuan 750021, China

**Keywords:** *Chlorella pyrenoidosa*, 3-fluorophenol, metabolic profiling, tolerance

## Abstract

This study investigates the growth tolerance mechanisms of *Chlorella pyrenoidosa* to 3-fluorophenol and its removal efficiency by algal cells. Our results indicate that *C. pyrenoidosa* can tolerate up to 100 mg/L of 3-fluorophenol, exhibiting a significant hormesis effect characterized by initial inhibition followed by promotion of growth. In *C. pyrenoidosa* cells, the activities of superoxide dismutase (SOD) and catalase (CAT), as well as the levels of malondialdehyde (MDA) and reactive oxygen species (ROS), were higher than or comparable to the control group. Metabolic analysis revealed that the 3-fluorophenol treatment activated pathways, such as glycerol phospholipid metabolism, autophagy, glycosylphosphatidylinositol (GPI)-anchored protein biosynthesis, and phenylpropanoid biosynthesis, contributed to the stabilization of cell membrane structures and enhanced cell repair capacity. After 240 h of treatment, over 50% of 3-fluorophenol was removed by algal cells, primarily through adsorption. Thus, *C. pyrenoidosa* shows potential as an effective biosorbent for the bioremediation of 3-fluorophenol.

## 1. Introduction

In recent years, the development of fluorinated chemical products has been extensive and rapid due to their “mimic effect” and “block effect” in living organisms. The van der Waals radius of fluorine atoms (1.35 Å) closely matches that of hydrogen atoms (1.09 Å), resulting in negligible molecular volume changes when hydrogen atoms are replaced by fluorine atoms. Consequently, fluorinated organic compounds exhibit significant biological effects such as enzyme inhibition, cell communication, and membrane transport [1]. These compounds are notably stable, resulting in prolonged biological half-lives and inhibiting normal metabolic processes. Due to these properties, fluorinated compounds are widely used in pharmaceuticals, disinfectants, herbicides, and wood preservatives [2,3]. These compounds enter the environment through industrial wastewater and agricultural activities primarily. In contaminated soils and sediments, the concentration of 3-fluorophenol can range from ng/L to µg/L [4]. In polluted surface and groundwater, monofluorophenol concentrations are typically lower, ranging from ng/L to µg/L [5]. The accumulation of fluorinated organic compounds in the environment poses potential hazards to both environmental organisms and human health [6,7,8,9,10,11]. These compounds are highly toxic, persistent, and bioaccumulative in general. For example, 3-fluorophenol can be degraded into 3-fluorocatechol and other small organic molecules by some fungi, bacteria, or photolysis [4,5,12]. However, the degradation process is challenging, and the pathways and products are complex.

In recent years, biological treatments have gained prominence for their effectiveness in pollutant remediation. Compared to chemical treatments like chlorine and ozone, which can produce toxic byproducts [2,13,14], biological treatments offer advantages such as high biodiversity, strong selectivity, and low secondary pollutant production. Consequently, they are essential for halogenated pollutant remediation [15,16].

The interaction between pollutants and biomaterials is complex, necessitating evaluations of removal efficiency, pollutant fate, and biological tolerance to assess biomaterial efficacy. While extensive research has focused on the bacterial treatment of halogenated pollutants, the issue of antibiotic resistance is becoming increasingly problematic [11,17,18,19]. In contrast, microalgae present numerous advantages as biomaterials for pollutant treatment. Microalgae are not targeted by antibiotics, thereby avoiding bacterial resistance issues and can be repurposed for various uses based on the pollutant removal mechanism as fuel, pigments, and fertilizers post-treatment [20,21,22,23]. Nevertheless, polluted environments can significantly inhibit microalgae growth and potentially cause irreversible cell damage, making high pollution tolerance crucial for effective application in pollution remediation.

*Chlorella* spp. are typical freshwater algae with wide distribution, rapid growth, and strong tolerance to extreme environments [24]. Studies have demonstrated that *Chlorella* spp. can effectively tolerate and remove diverse pollutants. For example, *C. vulgaris* 13-1 and *C. saccharophila* RNY efficiently remove pharmaceuticals such as caffeine, codeine, and ofloxacin [25,26]. *C. sorokiniana* can remove salicylic acid and paracetamol with efficiencies of 73% and 41–69%, respectively, primarily through biodegradation [27]. *Chlorella* sp. L38 shows good adaptability to 0.5 mg/L sulfadimethoxine, achieving a removal rate of around 88% through antioxidant enzyme secretion [17]. *C. pyrenoidosa* can remove ammonia nitrogen and total phosphorus from wastewater [11] and tolerate heavy metals such as Pb^2+^ [28] as well as various organophosphorus pesticide pollutants [29], indicating its good potential in environmental pollution control and ecotoxicological assessment. However, the potential of *C. pyrenoidosa* in the reduction in fluorinated organic pollutants remains underexplored. This study employs *C. pyrenoidosa* to investigate its tolerance and response mechanisms to 3-fluorophenol exposure, as well as its efficacy in removing 3-fluorophenol. By observing changes in biochemical indicators and cellular metabolic profiles, we aim to elucidate the mechanisms underlying *C. pyrenoidosa*’s tolerance and explore its potential applications in the remediation of 3-fluorophenol pollution.

## 2. Materials and Methods

### 2.1. Exposure of C. pyrenoidosa to 3-Fluorophenol

*C. pyrenoidosa* was obtained from the Institute of Hydrobiology, Chinese Academy of Sciences (Wuhan), catalog number FACHB-5. 3-Fluorophenol (CAS No. 372-20-3) with a purity of ≥98% was purchased from Shanghai Aladdin Biochemical Technology Co., Ltd. (Shanghai, China).

*C. pyrenoidosa* was cultured in a light incubator with a shaker, using Blue-Green 11 medium. The culture conditions were maintained at (25 ± 1) °C with a light intensity of 4000 lux and a light-to-dark ratio of 1:1. The rotation speed was set at 150 rpm. During the experiment, algae in the exponential growth phase were transferred to 250 mL Erlenmeyer flasks. The algae density was adjusted to approximately 1 × 10^5^ cells/mL using the culture medium, and 3-fluorophenol was added to achieve final concentrations of 10, 50, and 100 mg/L. Samples were collected every 48 h to measure algal biomass, 3-fluorophenol concentration, and biochemical indicators, continuing until 240 h of exposure.

Algal cells treated with 100 mg/L of 3-fluorophenol for 96 h were collected to measure biochemical indicators and metabolic profiles. Each experiment was repeated three times.

### 2.2. Determination of Algal Growth and Biochemical Indicators

The absorbance of algal solution at 680 nm was measured and was used to determine algal density.

To determine biochemical indicators, 10 mL of the algal solution was centrifuged at 4000 rpm for 10 min. The precipitates were collected and resuspended in 10 mL of phosphate buffer (0.01 mol/L, pH 7.8). The solution was then sonicated on ice for 15 min at a power of 200 W (ultrasonic 3 s and then rest 7 s) to break the cells. Reagent kits from Suzhou Keming Biotechnology Co., Ltd. (Suzhou, China) were used to test for superoxide dismutase (SOD, serial No.: SOD-1-W), catalase (CAT, serial No.: CAT-1-W), malondialdehyde (MDA, serial No.: MDA-1-Y), total soluble protein (TSP, serial No. BCAP-1-W), and reactive oxygen species (ROS, serial No.: ROS-1-Y). The values of TSP, ROS, SOD, CAT and MDA measured in mg/mL, ×10^−3^ u/s/μg prot, U/mg·prot, U/mg·prot, nmol/mg·prot, respectively. T-test analysis was performed on the control and 3-fluorophenol groups to analyze the significant differences.

### 2.3. Determination of Algal Metabolic Profile

The determination of algae metabolomics was conducted with reference to the method described by Li et al. [30]. After treating *C. pyrenoidosa* with 100 mg/L of 3-fluorophenol for 96 h, 20 mL of the algal solution was sonicated and then freeze-dried. The sample was re-dissolved in methanol. The control algal solution was also taken and treated for analysis. Ultra-high-performance liquid chromatography–tandem mass spectrometry (UPLC-MS/MS, Agilent 6546, Agilent Technologies, No.1 Yishun Avenue 7, Singapore, Singapore) was used for detection. Agilent Mass Hunter Profiler software was utilized for mass spectrometry data preprocessing, while Agilent Mass Profiler Professional (version 15.1) software was used to analyze the complex information content of the mass spectrometry data and conduct differential analysis. Differential metabolites were mapped to the Kyoto Encyclopedia of Genes and Genomes (KEGG) metabolite database (https://www.genome.jp/kegg, accessed on 13 March 2024) to identify corresponding metabolic pathways.

### 2.4. Determination of 3-Fluorophenol

To determine the concentration of 3-fluorophenol, 10 mL of the algal solution was centrifuged at 4000 rpm for 10 min. The supernatant was collected, and an equal volume of ethyl acetate was added, followed by vortex mixing for 1 min to extract 3-fluorophenol. The organic phase was collected for further analysis.

For detecting 3-fluorophenol in algal cells, cells from a 10 mL sample were collected and resuspended in 10 mL of phosphate buffer, then sonicated on ice. An equivalent volume of ethyl acetate was added to extract 3-fluorophenol. Additionally, a 10 mL sample of the algal solution was directly sonicated on ice to break the cells, and an equal volume of ethyl acetate was added to extract 3-fluorophenol. All organic phases were collected and prepared for GC-MS analysis.

The concentration of 3-fluorophenol was determined using Agilent 7890A/5975C gas chromatography–mass spectrometry (GC-MS). The chromatographic column used was HP-5, with a length of 30 m, an inner diameter of 250 µm, and a film thickness of 0.25 μm. The main GC-MS parameters were as follows: inlet temperature of 280 °C, ultrapure helium carrier gas flow of 1 mL/min, electron ionization (EI) source energy of 70 eV, ion source temperature of 230 °C, and collision energy of 5–50 V. The analysis utilized a programmed temperature rise, starting at 60 °C for 5 min, then increasing to 230 °C at a rate of 10 °C/min, and holding for 10 min.

## 3. Results and Discussion

### 3.1. Effects of 3-Fluorophenol on the Growth and Biochemical Indicators of C. pyrenoidosa

**Figure 1:**
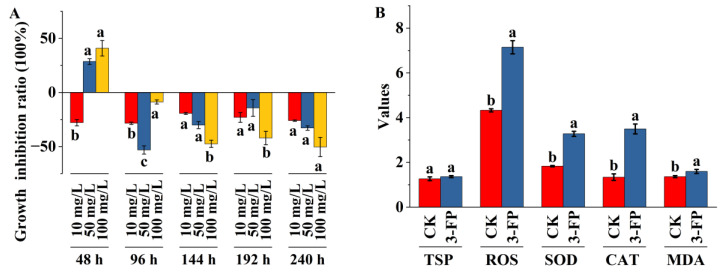
Effects of 3-fluorophenol on the growth and biochemical indicators of *Chlorella pyrenoidosa*. (**A**) Time–dose effect of 3-fluorophenol exposure on *C. pyrenoidosa* growth. (**B**) The biochemical indicators of *C. pyrenoidosa* treated by 100 mg/L 3-fluorophenol for 96 h. Note: a, b, and c indicate significant differences detected at *p* < 0.05. The values of TSP, ROS, SOD, CAT, and MDA refer to their levels in control (CK) or 3-fluorophenol (3-FP) samples, measured in mg/mL, ×10^−3^ u/s/μg prot, U/mg·prot, U/mg·prot, and nmol/mg·prot, respectively.

The time–dose effect of 3-fluorophenol on *C. pyrenoidosa* growth is shown in Figure 1A. Throughout the exposure cycle, 10 mg/L of 3-fluorophenol promoted the growth of algal cells. Initial concentrations of 50 and 100 mg/L of 3-fluorophenol initially inhibited the growth of *C. pyrenoidosa*, followed by a promotion effect. These beneficial effects at low doses of 3-fluorophenol and inhibitory effects at high doses conform to the hormesis model [31,32]. Mechanisms such as signal transduction, gene expression regulation, as well as overcompensation and adaptive responses, are attributed to this phenomenon [32,33].

Algal cells treated with 100 mg/L of 3-fluorophenol for 96 h were analyzed for biochemical indicators including TSP, SOD, CAT, MDA, and ROS (Figure 1B). Compared to the control group, the 3-fluorophenol treatment had little effect on the TSP content of algal cells, while levels of ROS, SOD, CAT, and MDA were significantly higher. TSP in algal cells are mostly enzymes involved in cellular metabolic activities, nutrient storage, and resistance to extreme environments. Changes in TSP content reflect changes in cellular metabolic levels [34]. The lack of significant difference in TSP between the 3-fluorophenol treated and control cells indicates normal protein metabolism.

ROS have dual roles in plant cells. They act as signaling molecules regulating various biological processes [35,36] and can also cause cellular damage due to their high reactivity [35]. To maintain homeostasis, cells rely on a complex antioxidant system to balance ROS production and scavenging. There is a complex interaction between ROS and antioxidant enzymes, with ROS inducing the expression of enzymes such as SOD and CAT in some cases [37]. Elevated levels of ROS, SOD, CAT, and MDA in the 3-fluorophenol treatment group indicate that exposure triggered a burst of intracellular ROS and modulated the cell’s defense mechanisms. MDA, a product of lipid peroxidation caused by polyunsaturated fatty acid oxidation, reflects cellular peroxidation damage levels [38]. The increase in MDA content in algal cells after 3-fluorophenol treatment indicates relatively weak ROS-induced cell membrane damage.

The above indicators show that after 96 h of exposure to 100 mg/L 3-fluorophenol, the metabolic balance of ROS in algal cells was disrupted. SOD and CAT were sensitive to ROS response, and the increase in enzyme activity effectively alleviated ROS oxidative damage. The cell metabolism was not significantly damaged. These results are consistent with the weak promoting effect of 100 mg/L 3-fluorophenol exposure on the growth of *C. pyrenoidosa* after 96 h (growth inhibition rate −8.85 ± 2.74%).

### 3.2. Effects of 3-Fluorophenol on the Metabolic Profiles of C. pyrenoidosa

**Figure 2:**
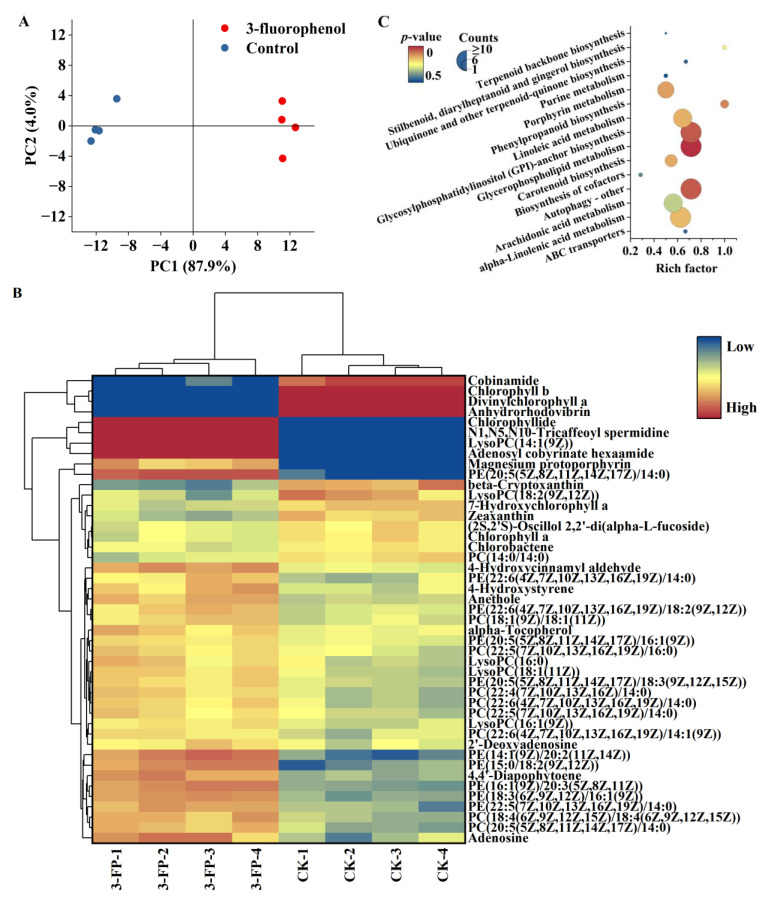
Effects of 3-fluorophenol on the metabolic profiles of *Chlorella pyrenoidosa*, (**A**) Principal component analysis. (**B**) Heatmap of differential metabolites. (**C**) Bubble diagram of differential metabolic pathways.

Algal cells treated with 100 mg/L 3-fluorophenol for 96 h were collected to investigate metabolic profiles. The results of principal component analysis (PCA) are shown in Figure 2A. The control group and treatment group samples were mapped to different regions, but each parallel sample was mapped to the same region, some of which were superimposed, indicating good intra-group parallelism and significant inter-group differences. This demonstrates that 3-fluorophenol exposure had a significant impact on the intracellular metabolites of *C. pyrenoidosa*.

Based on the criteria of fold change (FC) < −2 or >2 and *p*-value < 0.05, 172 differential metabolites were screened, with 80 metabolites upregulated and 92 downregulated. Enrichment of metabolic pathways was carried out using the KEGG database, with 46 metabolites enriched in glycerophospholipid metabolism, glycosylphosphatidylinositol (GPI)-anchored protein biosynthesis, autophagy, and other metabolic pathways. The heatmap of differential metabolites and bubble diagram of differential metabolic pathways are shown in Figure 2B,C.

Exposure to 3-fluorophenol resulted in an upregulation of various lipid metabolites in *C. pyrenoidosa*, including 9 types of phosphatidylcholines (PC) such as PC (14:0/14:0), 10 types of phosphatidylethanolamines (PE) such as PE (15:0/18:2 (9Z, 12Z)), and hemolytic phospholipids such as LysoPC (16:0). These metabolites were enriched in glycerophospholipid metabolism (*p* = 0.0012), GPI-anchored protein biosynthesis (*p* = 0.057), linoleic acid metabolism (*p* = 0.15), and alpha-linolenic acid metabolism (*p* = 0.16). Glycerophospholipids play a crucial role in cell membranes, ensuring stability, fluidity, and permeability. They are also essential for the optimal functioning of membrane proteins, receptors, and ion channels, and serve as repositories for second messengers and their respective precursors [39,40].

GPI anchoring modification, an important form of protein glycosylation in eukaryotic cells, plays a key role in signaling, cell growth, immune response, and cell development [41,42]. Autophagy is a cellular self-degradation mechanism in eukaryotes, important for cell self-protection [43,44]. Unsaturated fatty acids have antioxidant capacity [45], and their increased content helps maintain cellular metabolic stability. Lipid accumulation is often a survival strategy of microalgae to protect cells from oxidative stress under stressful conditions [46]. The upregulation of various lipid metabolites may be essential for *C. pyrenoidosa* to resist 3-fluorophenol stress.

Phenylpropanoids are bioactive secondary metabolites biosynthesized by plants from phenylalanine [47]. This biosynthesis is crucial for plant growth, development, reproduction, signaling, antioxidant activity, and responses to environmental stimuli, including tolerance and resistance against abiotic and biotic stresses [46,48,49,50]. After exposure to 3-fluorophenol, intermediate or final products of the phenylpropanoid pathway (*p* = 0.097) in *C. pyrenoidosa* cells, including anethole, 4-hydroxystyrene, and N1, N5, N10-tricaffeoyl spermidine, were upregulated, indicating activation of this pathway to help algal cells resist 3-fluorophenol stress.

Photosynthetic pigment content is a sensitive parameter for algal cells’ response to environmental stress. After exposure to 3-fluorophenol, levels of chlorophyll-a, chlorophyll-b, 7-hydroxychlorophyll-a, and cobinamide in *C. pyrenoidosa* cells were downregulated, while levels of chlorophyll, adenosyl cobyrinate hexaamide, and Mg-protoporphyrin were upregulated. These products were enriched in porphyrin metabolism (*p* = 0.13). The Mg-porphyrin ring is the core part of the chlorophyll molecule, undergoing reactions such as reduction and esterification to form chlorophyll-a; during this process, many chlorophyll-a precursor substances are generated, such as divinylchlorophyll-a. 7-hydroxychlorophyll-a is an intermediate product of the conversion between chlorophyll-a and -b [51,52]. Additionally, carotenoids, necessary photosynthetic pigments, bind to pigment–protein complexes on the membrane to exert their effects [53,54]. Exposure to 3-fluorophenol also resulted in the downregulation of anhydrohodovibrin, beta-cryptoxanthin, chlorobactene, and zeaxanthin levels in the carotenoid biosynthesis pathway (*p* = 0.14) of *C. pyrenoidosa*. It was observed that 3-fluorophenol significantly inhibited the growth of *C. pyrenoidosa* after 48 h of exposure (with an inhibition ratio of 40.80 ± 7.19%), but showed a weak promoting effect after 96 h. It can be inferred that 3-fluorophenol greatly interferes with the synthesis of chlorophyll and carotenoids. The downregulation of various intermediate products likely weakens the cells’ ability to absorb light, hindering photosynthesis. At 96 h, the photosynthetic system of algal cells is still inhibited.

For algal growth, an inhibitory effect is generally observed in the early treatment stages, with possible biomass promotion in later stages. The addition of organic pollutants may provide an organic carbon source, contributing to higher biomass. However, the tolerance of algal cells to pollutants shows a significant dose–effect relationship [55]. Generally, photosynthetic pigments, redox homeostasis, and DNA replication are vulnerable and can be easily disrupted by pollutants [56]. *Chlorella* sp. exhibits significant oxidative stress under cadmium, arsenic, copper, and zinc stress. It reduces oxidative stress by enhancing the activities of antioxidant enzymes such as SOD, CAT, glutathione reductase (GR), and ascorbate peroxidase (APX), thereby improving its bioremediation capacity [57,58,59]. *Chlorella* sp. can degrade 2,2′,4,4′-tetrabromodiphenyl ether (BDE-47) through adsorption, uptake, and metabolism, involving debromination, hydroxylation, and methoxylation, while BDE-47 can induce the production of hydrogen peroxide in the cell wall, plasma membrane, and chloroplast, enhancing the activity of antioxidant enzymes to alleviate oxidative stress [60]. Under 4-n-nonylphenol (4-n-NP) stress, *Chlorella* sp. exhibits disruptions in photosynthesis, carbohydrate metabolism, and protein synthesis, and mitigates the stress response through redox systems and energy metabolism [61]. Thus, it is inferred that algae cells exhibit various physiological and biochemical adaptation mechanisms under exogenous pollutant stress.

The structural characteristics of organic pollutants significantly impact the toxicological effects and bioremediation capacity of algal cells. For example, compared to 3-fluorophenol, *C. pyrenoidosa* exhibits a greater removal capacity for phenol and 4-fluorophenol, with a bioremoval rate exceeding 70%. The growth promotion under phenol and 4-fluorophenol stress is attributed to the accumulation of chlorophyll and glycerophospholipids, as well as reduced oxidative damage [30]. In our study, 3-fluorophenol treatment resulted in the downregulation of compounds related to porphyrin and carotenoid metabolism, indicating that the photosynthesis system is susceptible to interference. However, various lipids, which play pivotal roles in cell membrane integrity, autophagy, and GPI-anchored protein biosynthesis, were upregulated, suggesting a detoxification mechanism in algal cells. It has been reported that halogen substitution at the ortho-, meta-, and para-positions of phenol significantly affects the electron density and reactivity of the compounds [62]. Ortho- and para-substituted halophenols are more readily biodegradable than meta-substituted halophenols [11,63]. Consistent with these findings, our study found that *C. pyrenoidosa* has greater difficulty in removing 3-fluorophenol and exhibits more sensitive toxicity responses compared to phenol and 4-fluorophenol.

### 3.3. Removal of 3-Fluorophenol in the C. pyrenoidosa Solution

**Figure 3:**
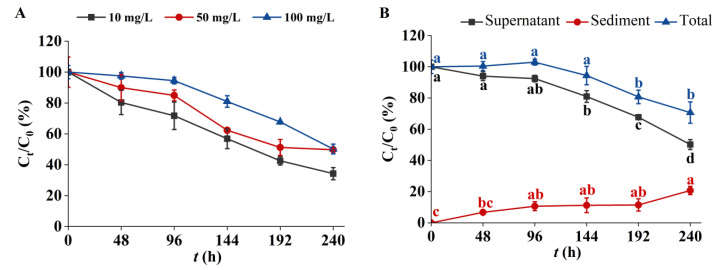
Removal efficiency of 3-fluorophenol in *C. pyrenoidosa* solution. (**A**) Removal efficiency of 3-fluorophenol at different initial concentrations. (**B**) Distribution of 100 mg/L 3-fluorophenol in algal solution. Note: a, b, and c indicate significant differences detected at *p* < 0.05 vs. C_0_/C_0_ of each group.

In the absence of algal cells, 3-fluorophenol could exist stably in the culture system (Supplementary Materials Figure S1). Figure 3A shows the removal efficiency of 3-fluorophenol by *C. pyrenoidosa*. The residual 3-fluorophenol in the algal solution decreased gradually over time. After 240 h of treatment, the residual ratios (C_t_/C_0_) of 3-fluorophenol at initial concentrations of 10, 50, and 100 mg/L were 34.26 ± 3.93%, 49.18 ± 0.64%, and 49.72 ± 3.18%, respectively. These results indicate that the removal efficiency of *C. pyrenoidosa* for 3-fluorophenol is strongly dependent on the initial concentration, with lower concentrations showing better reduction efficiency. This finding is consistent with Sharma et al. [46], who observed a similar decrease in the removal rate of sodium diclofenac by *C. sorokiniana* with increasing concentration.

Figure 3B illustrates the concentrations of 3-fluorophenol in the supernatant, sediment, and total algal solution. After 144 h of exposure, the content of 3-fluorophenol in the supernatant significantly decreased, while its content in the sediment gradually increased. During the initial 144 h, the concentration of 3-fluorophenol in the algal solution remained relatively unchanged but began to decrease gradually thereafter. Organic pollutants are adsorbed onto the surface of microalgae via various functional groups and are selectively transported into cells for biodegradation, which is a key mechanism in microalgae bioremediation of pollutants [64,65]. The surface of algal cells is rich in biomolecules such as lipids, polysaccharides, and amino acid residues that contain functional groups like hydroxyl and amino groups [65,66], Given that 3-fluorophenol is rich in aromatic rings and fluorine atoms, it is speculated that it is easily adsorbed on the surface of algal cells. The presence of 3-fluorophenol in the algal solution suggests that adsorption is the primary mechanism for its decrease during the early exposure stages. In the later stages (e.g., 192 or 240 h), the significant reduction in 3-fluorophenol content in the algal solution indicates that both adsorption and biodegradation contribute to its removal. Biological adsorption and biodegradation are distinct pathways, but differentiating between them quantitatively is challenging because adsorption is a preliminary stage of biodegradation [67,68]. Microalgae convert organic pollutants into simpler forms through enzymatic reactions such as hydrolysis, hydrogenation, hydroxylation, and glycosylation [11]. The biodegradability of a compound largely depends on its structural complexity; complex cyclic structures are generally more difficult to biodegrade than linear unsaturated structures [67]. This study demonstrates the degradation potential of *C. pyrenoidosa* for 3-fluorophenol.

## 4. Conclusions

This study developed a method for the biological treatment of 3-fluorophenol using *C. pyrenoidosa*. The algae effectively reduced 3-fluorophenol concentrations in water, achieving over 50% removal. Cell growth exhibited a hormesis effect at concentrations of 10–100 mg/L. The biochemical response of *C. pyrenoidosa* to 3-fluorophenol stress included stable levels of soluble proteins, enhanced activity of antioxidant enzymes (SOD and CAT), and the management of oxidative stress markers such as MDA and ROS. Metabolomics analyses revealed upregulation of lipid metabolism and key pathways such as glycerophospholipid metabolism, autophagy, and GPI-anchored protein biosynthesis, which contributed to the stabilization of cell membranes and enhanced antioxidant capacity. However, the downregulation of photosynthetic pigment metabolism pathways indicated vulnerability in the photosynthetic system under 3-fluorophenol exposure. These findings suggest that *C. pyrenoidosa* is a promising biomaterial for the bioremediation of water contaminated with 3-fluorophenol, due to its ability to maintain stable growth and significantly reduce pollutant levels.

## Data Availability

All relevant data are included in the paper or its Supplementary Materials.

## Supplementary Materials

The following supporting information can be downloaded at: https://www.mdpi.com/article/10.3390/metabo14080449/s1, Figure S1. Attenuation of 3-fluorophenol in BG11 medium.
