# Supplementary figures and images for: Tolerance Mechanisms and Removal Efficiency of Chlorella pyrenoidosa in Treating 3-Fluorophenol Pollution
Source: Metabolites. 2024 Aug 15;14(8):449. doi: 10.3390/metabo14080449 (PMC11356416; doi:10.3390/metabo14080449)

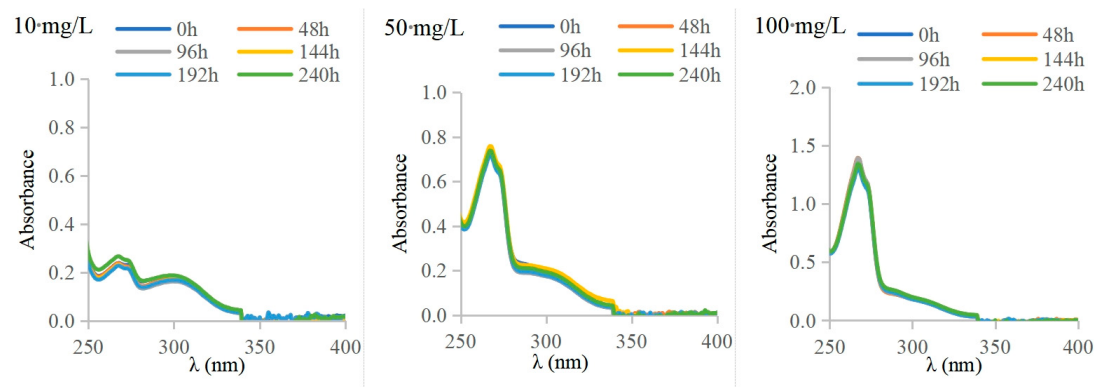

Figure S1. Attenuation of 3-fluorophenol in BG11 medium

Supplement: Supplementary file 1 [file metabolites-14-00449-s001.zip › metabolites-3144959-supplementary.pdf]
